# Hashimoto’s Thyroiditis and Dry Eye Disease

**DOI:** 10.3390/jcm14051710

**Published:** 2025-03-04

**Authors:** Karla Ranđelović, Tomislav Jukić, Andrea Tešija Kuna, Tamara Sušić, Milena Hanžek, Andrija Štajduhar, Zoran Vatavuk, Ivanka Petric Vicković

**Affiliations:** 1 Department of Ophthalmology, Sestre Milosrdnice University Hospital Center, 10000 Zagreb, Croatia; karla.randelovic@gmail.com (K.R.); zo.vatavuk@gmail.com (Z.V.); 2 School of Dental Medicine, University of Zagreb, 10000 Zagreb, Croatia; 3 Department of Oncology and Nuclear Medicine, Sestre Milosrdnice University Hospital Center, 10000 Zagreb, Croatia; tomislav.jukic@kbcsm.hr; 4 Department of Clinical Chemistry, Sestre Milosrdnice University Hospital Center, 10000 Zagreb, Croatia; andrea.tesija@kbcsm.hr (A.T.K.); tamara.susic@kbcsm.hr (T.S.); milena.hanzek@kbcsm.hr (M.H.); 5 Department of Medical Statistics, Epidemiology and Medical Informatics, School of Public Health “Andrija Štampar” and Medicine, University of Zagreb, 10000 Zagreb, Croatia; astajd@gmail.com

**Keywords:** Hashimoto’s thyroiditis, dry eye disease, biomarkers

## Abstract

Hashimoto’s thyroiditis (HT) is an autoimmune thyroid disease with characteristic lymphocytic infiltration and fibrosis. Chronic autoimmune changes that occur in the thyroid gland in HT may also affect the lacrimal gland. **Objectives**: This study aimed to analyze tear biomarkers and explore correlations between these biomarkers and clinical ocular parameters in patients with HT. **Methods**: A total of 150 participants were divided into three groups: HT (N = 50), non-HT DED (N = 50), and healthy controls (N = 50). The participants underwent a series of diagnostic tests for DED, including the Ocular Surface Disease Index, Tear Break-Up Time, Lid-Parallel Conjunctival Folds, Schirmer test without anesthetic, lissamine green and fluorescein staining. Tear samples were analyzed for cytokine and enzyme levels (interleukin 1β, tumor necrosis factor α, interleukin 6 (IL-6), interleukin 8, interleukin 10 (IL-10), interleukin 17A, matrix metalloproteinase 9 (MMP-9)) using ELISA and multiplex immunoassay. Statistical analyses were conducted to compare groups and assess biomarker correlations. **Results:** Dry eye disease was observed in more than half of the study group (27/50), with severe symptoms observed in 48.15% of the DED HT subgroup. IL-6 levels were significantly elevated in the DED HT subgroup compared to the non-HT DED group (*p* = 0.010), suggesting specificity for HT-associated DED. MMP-9 was elevated in both the HT and non-HT DED groups (*p* < 0.001) but lacked specificity for HT (*p* = 0.059). The DED HT subgroup exhibited significantly lower IL-10 levels (*p* = 0.008). Lissamine green staining and LIPCOF were significantly higher in the DED HT subgroup (*p* < 0.001). **Conclusions**: Dry eye disease is common in euthyroid HT patients without signs of TAO. This study highlights the potential role of IL-6. Lissamine green staining and LIPCOF are valuable diagnostic tools for assessing the ocular surface in DED HT patients.

## 1. Introduction

Hashimoto’s thyroiditis (HT) is an autoimmune thyroid disease. The incidence of HT is reported to be 0.3–1.5 cases per 1000 persons per year [1], although the prevalence is higher, 5% in the general population [2]. It is more common in women than in men, in a ratio of 4:1 to 10:1, depending on the literature [2,3,4]. The prevalence increases with age [5].

The disease is characterized by the infiltration of the thyroid tissue by lymphocytes, and the consequent deterioration of the thyroid due to immune processes [5,6]. In this disease, the patient’s immune system attacks the thyroid gland and creates antibodies against its components, which causes progressive fibrosis of the gland. Patients develop antibodies to thyroid peroxidase (TPO) and thyroglobulin (Tg), which leads to inadequate thyroid hormone production [1]. The main features of HT are autoimmunity, hypothyroidism, chronic inflammation, and, in some cases, goiter [7].

Autoimmune thyroid disease consists of two clinical presentations: Graves’ disease (GD) and HT [2]. The connection between GD and HT is known, as is that GD can evolve into HT and vice versa, which directly indicates that these two diseases have a very narrow pathophysiological path [8]. Cellular and humoral immunity are not separate processes; they overlap and work together even more powerfully. Activation of B cells and production of antibodies is the result of stimulation of T helper cells. Antibodies intensify the inflammatory process and, in this way, attract even more T cells to the thyroid gland, which closes the vicious circle of gland tissue destruction, chronic inflammation and the development of hypothyroidism [6,9,10]. Thyroid eye disease is most often described in GD [11], but it also refers to HT, which is rarely mentioned in the literature [12]. Elevated values of certain interleukins (IL-1β, IL-6, IL-8, IL-17, TNF-α) have been found in the tears of patients with GD [13,14], while dry eye disease (DED) is described with HT, without a clear immunological background, and the exact type of dry eye has not been confirmed so far [15,16,17].

Neufeld and Blizzard proposed the classification of polyglandular autoimmune syndrome (PAS) back in 1980. [18]. Within PAS, HT can be associated with other autoimmune diseases, such as Sjogren’s Syndrome (SS), and it explains the very common co-occurrence of both diseases, but the independent course of HT, without SS, is not sufficiently clarified [19]. PAS explains that hypothyroidism and antibodies affect multiple glands in HT, which can lead to dry eyes and mouth; PAS also points out that the problem is likely to be damage to the mucous membrane [20].

Dry eye disease, which can be hyposecretory or evaporative, is a multifactorial disease of tears and the ocular surface [21] that causes instability of the tear film, the key etiology of which is hyperosmolarity and inflammation with damage to the eye surface. Patients with a diseased thyroid gland have extremely high tear osmolarity; a theory suggests that it could be from excessive evaporation. Hyperosmolarity alone stimulates proinflammatory cytokines, including IL-1β, TNF-α, and MMP-9 [22,23]. These cytokines activate the MAPK cascade, which stimulates other cytokines [24,25,26]. IL-1 promotes an inflammatory response. IL-6 plays an important role in the regulation of immune reactions and can stimulate the production of antibodies. IL-10 is known for its anti-inflammatory properties. Dysfunction in the production or action of interleukins can contribute to the development of various diseases, including autoimmune disorders [27,28].

The disturbed immune response in HT opens space for newer diagnostic tests, and the detection of anti-inflammatory cytokines in the tear film is a non-invasive method that can have an additional role in monitoring. The aim of this study is to find the incidence of dry eye disease in patients with HT, to analyze the concentrations of biomarkers in tears (IL-1β, TNF-α, MMP-9, IL-6, IL-8, IL-10, IL-17A), and to analyze correlations between ocular surface parameters and tear biomarkers.

## 2. Materials and Methods

### 2.1. Study Design and Groups

This study was conducted at the Sestre milosrdnice University Hospital Center, in collaboration with Department of Ophthalmology, Department of Clinical Chemistry, Department of Oncology and Nuclear Medicine and School of Dental Medicine, University of Zagreb, from 2021 to 2024, in accordance with the Declaration of Helsinki and approved by the Ethics Committees of Sestre milosrdnice University Hospital Center and School of Dental Medicine, University of Zagreb.

This study included a total of 150 adult subjects divided into 3 groups: patients diagnosed with HT (N = 50), patients with dry eye disease (DED) without HT (non-HT DED) (N = 50), and age-matched healthy controls (N = 50).

After the confirmed diagnosis of HT (positive anti-TPO and anti-TG, in euthyroidsm, with a thyroid ultrasound indicating disease) in the Department of Oncology and Nuclear Medicine, the patients were referred to the Department of Ophthalmology. The following exclusion criteria were applied: positive anti-SSA and/or anti-SSB antibodies, positive Clinical Activity Score which refers to thyroid-associated ophthalmopathy (TAO), rheumatoid arthritis, systemic lupus erythematosus, scleroderma, polymyositis, dermatomyositis, primary biliary cholangitis, hematochromatosis, multiple sclerosis, Sjögren Syndrome (SS), diabetes, malignant diseases, amyloidosis, leprosy, sarcoidosis, tuberculosis, pregnant and women who are breastfeeding, smokers, contact lenses users, people who have acute allergic and viral conjunctivitis, people who had previous eye surgeries, the presence of pterygium, treated with radiotherapy of the head and neck in the last year, the use of corticosteroids (local or systemic) in the last year, people who suffer from hepatitis C, people who have had a recent infection with the Epstein–Barr virus, and people taking medications or drops that can cause dryness.

After the aforementioned exclusion criteria were applied, the eligible patients with HT needed to fill out the OSDI questionnaire for the detection of symptoms of dry eyes (OSDI: normal, mild, moderate, severe) [21]. After that, objective tests were performed to assess ocular surface (TBUT, Schirmer test without anesthetic, staining of the cornea and conjunctiva with fluorescein and lissamine green dye, LIPCOF) [29,30], and a tear sample was taken using microcapillaries (Drummond Scientific Company, SAD, Broomall, PA, USA) [31]. Tears were stored at −80 degrees Celsius until analysis. DED was defined according to the TFOS DEWS II criteria (OSDI ≥ 13 + 1 positive diagnostic test (TBUT < 10 s or ocular surface staining: >5 corneal spots, >9 conjunctival spots) [29,31].

The group with DED underwent the same test procedure as the HT group. These are patients with dry eyes that match the age and the gender of the examined HT group and have an etiological factor that causes dry eyes; HT and SS were excluded, as were patients those using antiglaucoma therapy, pregnant patients, people who have acute allergic or viral conjunctivitis, people who have had previous eye operations, and people who tested positive for the presence of pterygium. These patients were selected from the outpatient clinic referred to correction of refractive error with glasses or for preoperative treatment before cataract surgery.

The group of healthy patients was subjected to the same test procedure as the HT group. This group matches the age and gender of the studied HT group, they do not have any etiological factor (listed in the exclusion criteria of the HT group) that causes dry eyes, and HT is excluded.

### 2.2. Tear Fluid Analysis

An immunological analysis of tears, which included MMP-9, IL-1β, TNFα, IL-6, IL-8, IL-10, and IL-17A, was performed at the Department of Clinical Chemistry. MMP-9 concentration was determined using an ELISA test (Invitrogen, Thermo Fischer Scientific Inc., Waltham, MA, USA) using a VirCLIA analyzer (Vircell, Granada, Spain). The concentrations of IL-1β, TNFα, IL-6, IL-8, IL-10, and IL-17A in the tear samples were determined with the bead-based multiplex assay (LEGENDplex customized Human Inflammation Panel 1, BioLegend, San Diego, CA, USA). This assay uses a mixture of fluorescence-encoded capture beads of different sizes, covalently conjugated to each interleukin-specific antibody. It is based on the principle of sandwich immunoassay and allows for simultaneous quantification of six different interleukins from 1 sample aliquot. The final immunocomplexes, comprising tear-derived interleukins sandwiched between antibody-labeled capture beads and fluorescently labeled detection antibodies, provide fluorescent signal intensities proportional to the amount of bound interleukins. The quantification of the bead-captured interleukins was performed using flow cytometer—the ZE5 Cell Analyzer (4 lasers, 24-color, Bio-Rad, Hercules, CA, USA) and LEGENDplex data analysis software (https://www.biolegend.com/en-ie/immunoassays/legendplex/support/software, accessed on 28 February 2025), where the concentration (pg/mL) of each interleukin was obtained from the standard curve created by plotting the mean fluorescence intensity (MFI) versus the concentration.

### 2.3. Data Analysis

Statistical analysis was conducted to evaluate the differences between three groups: HT, non-HT DED, and the healthy group. Descriptive statistics were used to summarize baseline characteristics, with results presented as means ± standard deviations or median with interquartile range (IQR). Group comparisons were performed using one-way ANOVA for continuous variables, such as age and biomarker levels, with post hoc tests to identify pairwise differences. Non-parametric variants of statistical tests were applied for non-normally distributed data. Correlations between tear biomarkers and clinical parameters were assessed using Spearman’s rank correlation coefficients. Biomarker levels were compared across subgroups using Mann–Whitney U tests. Statistical significance was set at *p* < 0.05, and adjustments for multiple comparisons were made using the Holm–Bonferroni correction.

## 3. Results

A total of 40 men and 110 women participated in this study. There were no significant differences in mean age and sex distribution among the analyzed groups.

The results of testing ocular surface dryness are shown in Table 1. All diagnostic methods used evaluated HT-related ocular surface dryness, except for the Schirmer test without anesthetic. In the HT group, 86% participants had a positive TBUT test. The LIPCOF test was significant and specific for the HT group.

**Figure 1:**
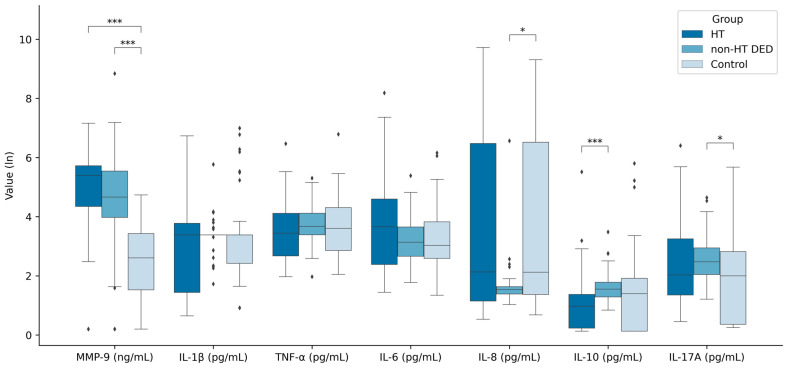
Comparison of tear cytokine levels and matrix metalloproteinase 9 in Hashimoto’s thyroiditis (HT) group, dry eye disease group without Hashimoto’s thyroiditis (non-HT DED), and healthy subjects. Statistical analysis was conducted using Dunn post hoc tests following significant results from Kruskal–Wallis tests for non-normally distributed data, where one asterisk (*) represents *p* < 0.05, and three (***) represent *p* < 0.001, for the following comparisons: HT vs. non-HT DED, HT vs. control, and non-HT DED vs. control.

Tear biomarkers across the three groups are illustrated in Figure 1. In comparison to the healthy group, MMP-9 (*p* < 0.001), IL-6 (*p* = 0.333), IL-8 (*p* = 0.329), IL-17 (*p* = 0.303) were elevated in the HT group; however, statistical significance was observed only for MMP-9. Additionally, MMP-9 levels were significantly higher in the DED group compared to the healthy group (*p* < 0.001). However, MMP-9 was not specific to the HT group alone (*p* = 0.059).

In the HT group, IL-10 levels were significantly higher compared to the non-HT DED group (*p* < 0.001), and they were also higher in the healthy group, but without statistical significance (*p* = 0.072). In the non-HT DED versus the control group, statistically significant differences were observed for IL-8 and IL-17A, with higher cytokine concentrations in the control group.

The HT group was further divided into two subgroups based on TFOS DEWS II criteria: the non-DED HT subgroup (N = 23) and the DED HT subgroup (N = 27). Among the HT patients, 54% had DED.

From the diagnostic tests assessing ocular surface, OSDI, lissamine green staining and LIPCOF were statistically significant in DED HT subgroup compared to the non-HT DED group. Patients with DED HT reported severe dry eye symptoms, with a mean OSDI score of 33.41 ± 14.45 compared to 28.10 ± 13.64 in the non-HT DED group, indicating moderate symptoms. In the DED HT subgroup, 33.33% of patients had mild symptoms, 18.52% moderate, and 48.15% severe symptoms.

TBUT was low in both the DED HT subgroup (4.89 ± 2.48) and in the non-HT DED group (3.56 ± 2.09), with no statistical significance among the subgroup and the group (*p* = 0.055). All diagnostic tests in both the DED HT subgroup and the non-HT DED group indicated dry ocular surfaces, except for the Schirmer test without anesthetic (Table 2).

The tear biomarkers medians are shown in Table 3. By comparing tear biomarkers in the DED HT subgroup and the non-HT DED group, IL-6 and IL-10 emerged as key markers. IL-6 levels were significantly higher in the DED HT subgroup compared to the non-HT DED group (*p* = 0.010). Conversely, the non-HT DED exhibited significantly higher IL-10 levels compared to DED HT subgroup. The median of MMP-9 (216.6 (69.9–328.2)) and IL-6 (68.6 (24.8–138.1)) was higher for the DED HT subgroup compared to the non-HT DED and control group. IL-17A was higher in the DED HT subgroup compared to the other two groups, though the difference in IL-17A levels was not statistically significant. There was only statistical significance for IL-17A in the non-HT DED group compared to control.

**Figure 2:**
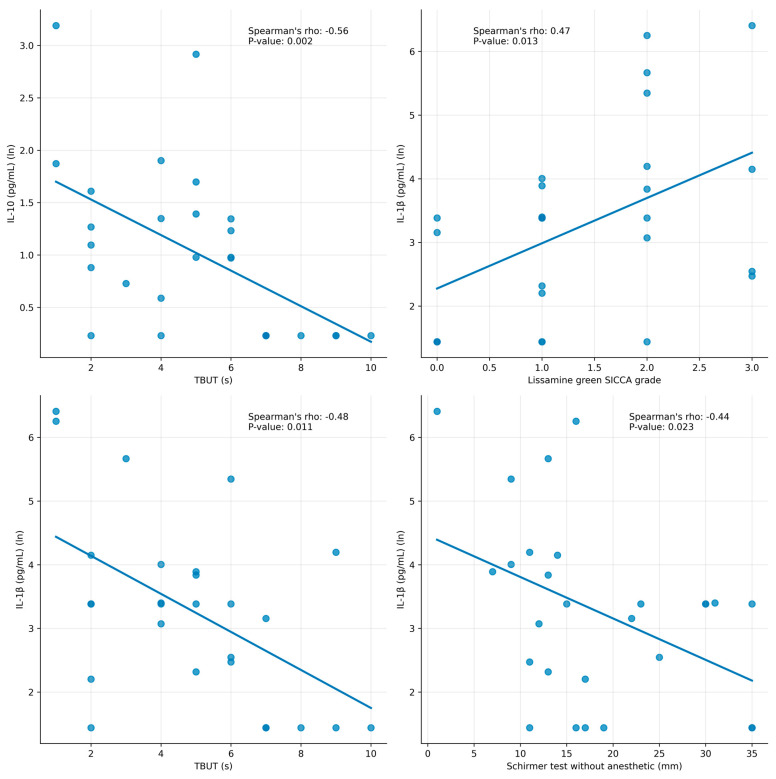
Correlations between biomarkers levels in tears and clinical parameters in Hashimoto’s thyroiditis subgroup with dry eye disease (DED HT). Spearman’s correlations were calculated.

Next, we analyzed the following correlations between tear biomarkers and ocular surface parameters (OSDI, Oxford scale, lissamine green staining, TBUT, Schirmer test without anesthetic and LIPCOF) in the DED HT subgroup (Figure 2). In this subgroup, only IL-10 (r = −0.56, *p* = 0.002) and IL-1β (r = −0.48, *p* = 0.011) were negatively correlated with TBUT. IL-1β had a statistically significant negative correlation with the Schirmer test without anesthetic (r = −0.44, *p* = 0.023), but a statistically positive correlation with lissamine green staining (r = 0.47, *p* < 0.013). Other biomarkers did not show significant correlations with ocular parameters in the DED HT subgroup.

In our cohort of euthyroid HT patients without TAO, those with DED HT exhibited higher median levels of thyroid autoantibodies compared to those with non-DED HT. Specifically, the median anti-TPO level in the DED HT group was 1301 kIU/L (range: 424.5–1301) versus 701.2 kIU/L (range: 253.6–1301) in the non-DED HT group, while the median anti-TG level was 7 kIU/L (range: 1.1–93.6) compared to 4.9 kIU/L (range: 1.2–36.6). No significant correlation was found between these antibody titers and the specific DED parameters.

## 4. Discussion

In this research, mostly women (73.3%) were included, which corresponds to the frequency of HT in the literature [2].

Although GD is more researched, both autoimmune thyroid diseases (AITDs) are associated with ocular symptoms. Among these, dryness is frequently mentioned as a symptom. The incidence of dry eye among AITD patients varies depending on the diagnostic criteria used, ranging from 27–96% with single diagnostic tools [32] to 45–85% according to TFOS DEWS II [33]. Similarly, Kan et al. demonstrated a significant overlap between HT and DED, further supporting the notion that DED is a frequent complication of HT [17]. In our study group, we analyzed the presence of dry eye in patients with HT who were euthyroid and without TAO. Dry eye disease was observed in more than half of the study group (27/50). Our patients were primarily from the Department of Oncology and Nuclear Medicine, and DED was not their primary symptom. Despite the undiagnosed DED, analyzing OSDI, 51.85% of HT patients had mild-to-moderate dry eye, while 48.15% had severe DED. Dry eye disease diagnosis was made for the first time at our ophthalmological examination. It should also be noted that our study group was young, as the mean age in all three groups examined was below 40 years. The younger population generally displays lower awareness of DED and its associated symptoms. From clinical observations, patients with AITD and TAO are more prone to the occurrence of DED, whereas patients without TAO are often unaware of DED-related issues.

Our results, particularly from the lissamine green conjunctival staining test, clearly indicate a mucous deficiency in DED associated with HT. This finding supports the role of lacrimal gland dysfunction in this condition [34]. Most of our HT patients had a positive TBUT (6.36 ± 4.63 s), which was also reported by Altin Ekin et al. [16]. LIPCOF (2.15 ± 1.35) was significant and specific for the HT group; that has been reported in dry eyes of patients with autoimmune rheumatic diseases [35], but not yet in AITDs [36]. TBUT was negatively correlated with IL-10 and IL-1β, and lissamine green was positively correlated with IL-1β.

While the elevated levels of anti-TPO and anti-TG do not consistently predict the progression to hypothyroidism or directly reflect overall disease severity [37], recent research indicates a positive correlation between the severe symptoms in HT patients and elevated thyroid antibody levels; especially, higher titers of anti-TPO are associated with more severe clinical symptoms [38]. Although findings in our study suggest a trend towards an association between elevated antibody levels and the presence of DED, we did not observe a significant correlation between these titers and the DED parameters.

The relationship between GD and DED has been well documented in the literature, with DED being frequently associated with TAO [39]. TAO is an autoimmune condition characterized by the binding of specific autoantibodies to orbital fibroblasts, which release chemokines, leading to lymphocytic infiltration. Research on the mechanisms underlying DED in TAO suggests multiple contributing factors, including reduced tear production due to the lacrimal gland being a target of thyroid-stimulating hormone (TSH) [40,41,42], and an increase in palpebral fissure height, which accelerates tear evaporation [43]. Furthermore, recent findings indicate that the pathogenesis of Graves’ orbitopathy is also influenced by immunological factors, such as T-cell-mediated inflammation and TSH receptor expression in the acinar cells of impaired lacrimal glands [40,41].

It has been reported that TAO results from T-lymphocyte-mediated stimulation of orbital cells. Expression of THS receptors in GD patients has been reported, and the lacrimal gland is recognized as a target organ for TSH [40,41,42]. Ludgate et al. investigated TSH receptor expression in lacrimal gland cells, which, despite being receptors, are also considered antigens shared between the thyroid gland and extrathyroidal sites affected by GD [44]. Similarly, Eckstein et al. explored the presence of TSHR in the lacrimal gland of TAO patients and its impact on ocular surface conditions [42]. Inoue et al. reported a high prevalence of obstructive meibomian gland disease among DED patients with GD TAO, as well as lacrimal gland swelling observed in MRI findings, which was accompanied by lower Schirmer test values [13].

Given that the lacrimal glands can be affected by TAO, the analysis of inflammatory cytokines in the tears of patients with GD and TAO has been the subject of many studies [14,45,46,47,48,49]. Cytokines seem to play a crucial role in disease development by participating in the induction and effector phases of immune interactions between T and B cells, macrophages, orbital fibroblasts, and orbital adipocytes [45,46,50]. Kishazi et al. reported a significant elevation in IL-10, IL-12p70, IL-6, IL-13 and TNF-α in GD TAO patients compared to the control group, and L-10, IL-12p70 and IL-8 levels were significantly elevated in TAO regardless of the activity of the disease (CAS). IL-13, IL-6, and TNF-α appeared to be elevated in inflammatory TAO patients who were in the active phase of TAO (CAS > 3) compared to the controls. The findings obtained for TNF-α, IL-6, IL-8 IL-10 and IL-13 confirmed those of previous studies on tears [14,45,51] but also on serum [45,46,47,48,52]. Mandic Juri et al. reported statistically significant higher levels of IL-6 in the GD group without TAO compared to the healthy group. The IL-10 and IL-1α levels were higher in the control group than in the GD group without TAO [53]. In our study, we also found elevated IL-6 levels and reduced IL-10 levels. Ujhelyi et al. [45] aimed to analyze the cytokine composition in the tears of patients with GD, both with and without orbitopathy. They showed that the levels of IL-1β, IL-6, IL-13, IL-17A, IL-18, and TNF-α were significantly higher in patients with GD compared to the controls, and they also highlighted IL-6 in tears as a potential indicator of disease activity in GD. However, none of the examined cytokines successfully differentiated between these two groups.

Unlike GD, where numerous studies have analyzed the presence of dry eye and the cytokine profile of tears, only a few studies have examined the occurrence of dry eye in HT. However, no study has analyzed the cytokine profile of tears in patients with HT.

Analyzing biomarkers in patients with HT and in control groups, our goal was to determine whether there are specific biomarkers characteristic of our study group. Among the tear biomarkers measured, MMP-9 was significantly elevated in both the HT and DED groups (*p* < 0.001) compared to the healthy group, but it lacked specificity for HT alone (*p* = 0.059). This suggests that both groups exhibit pronounced tear osmolarity, a finding consistent with the studies by Kook et al. and Pflugfelder et al., which identified MMP-9 as a marker of ocular surface inflammation in dry eye associated with systemic autoimmune diseases. These studies also indicate that local therapy with doxycycline, azithromycin, dexamethasone, and cyclosporine A may inhibit MMP-9 and thereby influence biomarker levels [54,55]. However, our study highlights that while MMP-9 is elevated, it lacks specificity for HT, echoing the findings of Lee et al. in Sjögren’s syndrome (SS) [56], where MMP-9 was a general marker of inflammation rather than disease-specific.

In our study, we did not find a significant difference between the DED HT group and the control groups in the levels of IL-1β, TNFα, IL-8, and IL-17A. The concentration of IL-6 was statistically significantly elevated in the DED HT group compared to both control groups, while IL-10 was lower in the DED HT group compared to the control groups.

IL-6 emerged as a specific biomarker for HT-associated DED in our study, a finding that aligns with the work of Luo et al., who demonstrated the critical role of IL-6 in triggering and sustaining inflammatory cascades in dry eye disease [26]. Elevated IL-6 levels in HT-associated DED also align with studies on GD, where inflammatory cytokines like IL-6 were similarly implicated in ocular surface pathology [14]. IL-6 is a proinflammatory cytokine that reduces tear production and alters tear quality, providing critical information about eye surface status [28]. It appears early in the inflammatory cascade, triggering the activation of other immune responses, including IL-8, which mobilizes neutrophils to address tissue damage [27,28,57]. Elevated IL-6 values were found in patients with HT who were euthyroid and did not present signs of TAO. This suggests that, even when the autoimmune disease is well controlled and ocular symptoms may not be severe, IL-6 serves as an indicator of ongoing ocular surface inflammation and damage. These findings may also suggest practical implications for the early diagnosis and management of HT-associated DED. Despite these results, its diagnostic utility should be interpreted with caution due to its broad involvement in various inflammatory pathways [58].

IL-17A, a marker of chronic inflammation typical of autoimmune processes, was higher in the DED HT subgroup compared to non-HT DED and healthy individuals, but the difference was not statistically significant (*p* = 0.824). Our results indicate that our HT group includes patients in various stages of autoimmune disease progression.

Additionally, the low IL-10 levels observed in the DED HT subgroup indicate an uncontrolled inflammatory state, further supporting the need for early therapeutic intervention. In our study, IL-10 tear levels were found to be lower than in the control group, but the difference was not statistically significant. IL-10 is an anti-inflammatory cytokine predominantly associated with the action of Th2 helper T cells. Generally, it reduces tissue inflammation and stimulates B cell differentiation. This finding contrasts with SS, where IL-10 levels are typically elevated compared to the control group [56,59,60]. Akpek et al. [61] reported a lower concentration of IL-10 in the SS DED group compared to the control groups (non-DED and control). A limitation of their study may be that patients in the control group did not undergo a specific workup for SS beyond medical history and ophthalmologic examination. Although IL-10 has been found in tears, a study also reports elevated levels in the salivary glands and serum of SS patients [62]. These findings support the association of the TH-2 response in SS and suggest that IL-10 influences inflammatory processes.

Tear levels, as well as serum levels of IL-10, were found to be higher in patients with TAO than in healthy subjects [46,47,51]. Takeoka et al. showed elevated levels of serum IL-10 in GD patients during remission or in HT patients [63]; in contrast, Mandic Juri et al. found a decreased concentration of tear IL-10 in hyperthyroid patients without TAO [53], which was also shown by the results of our study. When comparing tear biomarkers between GD and HT, IL-6 was the only marker detected in HT, unlike in GD. This finding may suggest that the two diseases are at different stages. Type 1 regulatory T (Tr1) cells are the primary producers of IL-10, which suppresses autoimmune processes [64]. Additionally, B cells have been shown to be able to provide IL-10-mediated protection against autoimmunity in mice [65]. These findings align with various studies suggesting that IL-10 plays a protective role in several animal models of autoimmune diseases [66,67]. However, some reports indicate that IL-10 worsens antibody-mediated autoimmune diseases [68]. Thus, it remains uncertain whether IL-10 production primarily regulates autoimmune processes or contributes to disease progression. Introducing anti-inflammatory treatments targeting IL-6 and MMP-9 could potentially mitigate disease progression and prevent long-term damage to the ocular surface.

Autoimmune thyroid disease comprises two main clinical presentations: GD and HT. However, these disorders represent opposite ends of the same underlying cause. Patients with HT have a five-times-higher risk of developing another autoimmune disease [69,70]. Boalert et al. reported that 14.3% of HT patients had another autoimmune disorder, with rheumatoid arthritis being the most common associated disease [71]. SS and AITD share a common pathophysiological mechanism, and the coexistence of SS and HT has been documented. Complications arising from SS-related DED can cause severe ocular damage. However, patients often experience significant delays in diagnosis, with some studies indicating that it can take up to seven years from symptom onset to diagnosis [72]. Early diagnosis of HT-related DED is important so that patients can be effectively treated and further complications can be prevented.

However, certain limitations should be considered when interpreting our findings. One of the limitations of our study is the relatively small sample size. We did not consider the duration of the disease, which could affect DED severity. In our study, we analyzed only patients in euthyroidism; further investigations could include patients in different stages of the disease. Comparisons with other studies are not straightforward due to differences in classification criteria, sampling methods, and analytical methods. Many studies have identified cytokines involved in diseases, but few studies report negative results, which are crucial for elucidating etiology. Another important issue when comparing with other studies is the absence of a positive control group, making it difficult to draw conclusions about biomarker specificity [49,53].

## 5. Conclusions

Dry eye disease is common in HT patients who are euthyroid and do not show signs of TAO. Lissamine green staining and LIPCOF are important ocular parameters in the observed group. This study highlights the potential role of IL-6 as a biomarker for HT-associated DED, while MMP-9 was found to be elevated in both the HT and the non-HT DED group, suggesting its role in ocular surface inflammation but lacking specificity for HT. TBUT negatively correlates with IL-10 and IL-1β, while lissamine green staining positively correlates with IL-1β. Early diagnosis of HT-related DED is important so that patients can be effectively treated and prevent further complications.

## Figures and Tables

**Table 1:** Demographics and clinical characteristics of Hashimoto’s thyroiditis group, dry eye disease group without Hashimoto’s thyroiditis, and healthy subjects, shown as mean ± standard deviation.

Characteristics	Hashimoto’s Thyroiditis (HT)	Dry Eye Disease Without Hashimoto’s Thyroiditis (non-HT DED)	Control	*p* Value (HT vs. non-HT DED)	*p* Value (HT vs. Control)	*p* Value (non-HT DED vs. Control)
Number of patients	50	50	50			
Age (years) ± SD	39.22 ± 11.20	38.78 ± 10.49	35.08 ± 9.60	0.834	0.148	0.157
Sex (M/F)	10/40	15/35	15/35	0.248	0.248	1.000
OSDI (0–100) ± SD	21.78 ± 16.85	28.10 ± 13.64	3.22 ± 3.94	**0.014**	**<0.001**	**<0.001**
Oxford scale (0–5) ± SD	1.56 ± 1.05	1.78 ± 1.00	0.10 ± 0.30	0.323	**<0.001**	**<0.001**
Lissamine green SICCA grade (0–4) ± SD	1.20 ± 0.95	0.86 ± 0.78	0.04 ± 0.20	0.105	**<0.001**	**<0.001**
TBUT (s) ± SD	6.36 ± 4.63	3.56 ± 2.09	12.94 ± 3.62	**0.002**	**<0.001**	**<0.001**
Schirmer test without anesthetic (mm) ± SD	18.52 ± 9.51	16.40 ± 9.13	21.48 ± 8.61	0.265	0.204	**0.018**
LIPCOF grade (0–3) ± SD	1.80 ± 1.39	1.04 ± 0.95	0.24 ± 0.43	**0.010**	**<0.001**	**<0.001**

OSDI—Ocular Surface Disease Index; SD—standard deviation; SICCA—Sjogren’s International Collaborative Clinical Alliance; TBUT—Tear Break-Up Time; LIPCOF—Lid Parallel Conjunctival Folds; HT—Hashimoto’s thyroiditis; DED—dry eye disease; non-HT DED—Dry eye disease without Hashimoto’s thyroiditis. Statistical analysis was conducted using Dunn post hoc tests following significant results from Kruskal–Wallis tests for non-normally distributed data. *p*-values were adjusted for multiple comparisons using the Holm–Bonferroni correction. Significant *p*-values (*p* < 0.05) are shown in bold.

**Table 2:** Clinical characteristics of Hashimoto’s thyroiditis subgroup with dry eye disease, dry eye disease group without Hashimoto’s thyroiditis, and healthy subjects, shown as mean ± standard deviation.

Characteristics	Hashimoto’s Thyroiditis with Dry Eye Disease (DED HT)	Dry Eye Disease Without Hashimoto’s Thyroiditis (non-HT DED)	Control	*p* Value (DED HT vs. non-HT DED)	*p* Value (DED HT vs. Control)	*p* Value (non-HT DED vs. Control)
Number of patients	27	50	50			
OSDI (0–100) ± SD	33.41 ± 14.45	28.10 ± 13.64	3.22 ± 3.94	**0.049**	**<0.001**	**<0.001**
Oxford scale (0–5) ± SD	1.74 ± 1.00	1.78 ± 1.00	0.10 ± 0.30	0.838	**<0.001**	**<0.001**
Lissamine green SICCA grade (0–4) ± SD	1.41 ± 0.95	0.86 ± 0.78	0.04 ± 0.20	**<0.001**	**<0.001**	**<0.001**
TBUT (s) ± SD	4.89 ± 2.48	3.56 ± 2.09	12.94 ± 3.62	0.055	**<0.001**	**<0.001**
Schirmer test without anesthetic (mm) ± SD	18.15 ± 9.19	16.40 ± 9.13	21.48 ± 8.61	0.417	0.246	**0.016**
LIPCOF grade (0–3) ± SD	2.15 ± 1.35	1.04 ± 0.95	0.24 ± 0.43	**<0.001**	**<0.001**	**<0.001**

OSDI—Ocular Surface Disease Index; SD—standard deviation; SICCA—Sjogren’s International Collaborative Clinical Alliance; TBUT—Tear Break-Up Time; LIPCOF—Lid Parallel Conjunctival Folds; HT—Hashimoto’s thyroiditis; DED—dry eye disease; non-HT DED—Dry eye disease without Hashimoto’s thyroiditis; DED HT—subgroup with dry eye disease and Hashimoto’s thyroiditis. Statistical analysis was conducted using the Holm post hoc test following significant results from ANOVA tests for normally distributed data. For normally distributed variables, parametric tests were applied where appropriate. Significant *p*-values (*p* < 0.05) are shown in bold.

**Table 3:** Tear biomarker levels in Hashimoto’s thyroiditis subgroup with dry eye disease (DED HT), dry eye disease group without Hashimoto’s thyroiditis (non-HT DED), and healthy subjects, shown in median and interquartile range.

Cytokine	Hashimoto’s Thyroiditis with Dry Eye Disease (DED HT) Med (IQR)	Dry Eye Disease Without Hashimoto’s Thyroiditis (non-HT DED) Med (IQR)	Control Med (IQR)	*p* Value (DED HT vs. non-HT DED)	*p* Value (DED HT vs. Control)	*p* Value (non-HT DED vs. Control)
MMP-9 (ng/mL)	216.6 (69.9–328.2)	104.9 (52.3–253.9)	12.5 (3.6–29.8)	0.109	**<0.001**	**<0.001**
IL-1β (pg/mL)	28.5 (8.6–50.9)	28.5 (28.5–28.5)	28.5 (10.3–28.5)	1.000	1.000	0.846
TNF-α (pg/mL)	25.0 (13.5–53.4)	38.2 (28.5–60.3)	35.8 (16.4–73.3)	0.246	0.463	0.461
IL-6 (pg/mL)	68.6 (24.8–138.1)	22.1 (13.3–37.6)	19.7 (12.3–44.9)	**0.010**	**0.010**	0.995
IL-8 (pg/mL)	6.1 (2.2–510.2)	3.6 (3.0–4.1)	7.4 (2.9–675.8)	0.482	0.482	**0.022**
IL-10 (pg/mL)	1.7 (0.3–2.9)	3.7 (2.6–4.9)	3.0 (0.1–5.8)	**0.008**	0.163	0.163
IL-17A (pg/mL)	13.0 (2.1–53.4)	10.9 (6.7–18.0)	6.4 (0.5–15.7)	0.824	0.085	**0.022**

MMP-9—matrix metalloproteinase 9; IL-1β—interleukin 1β; TNF-α—tumor necrosis factor α; IL-6—interleukin 6; IL-8—interleukin 8; IL-10—interleukin 10; IL-17A—interleukin 17A; HT—Hashimoto’s thyroiditis; DED—dry eye disease, non-HT DED—dry eye disease without Hashimoto’s thyroiditis; DED HT—subgroup with dry eye disease and Hashimoto’s thyroiditis, Med—median; IQR—interquartile range. Statistical analysis was conducted using Dunn post hoc tests following significant results from Kruskal–Wallis tests for non-normally distributed data. Statistically significant *p* values between groups are shown in bold.

## Data Availability

The raw data supporting the conclusions of this article will be made available by the authors on request.
